# *Escherichia coli* with penicillin-binding protein 3 insertions: implications for newer β-lactam/β-lactamase inhibitor combinations

**DOI:** 10.1128/aac.01077-26

**Published:** 2026-09-16

**Authors:** Johann D. D. Pitout, Gisele Peirano, Yasufumi Matsumura, Marleen Kock, Liang Chen

**Affiliations:** 1 Cumming School of Medicine, University of Calgary 2129, Calgary, Alberta, Canada; 2 Alberta Precision Laboratories, University of Calgary 590576, Calgary, Alberta, Canada; 3 University of Pretoria 56410, Pretoria, Gauteng, South Africa; 4 Kyoto University Graduate School of Medicine 12918, Kyoto, Japan; 5 School of Pharmacy and Pharmaceutical Sciences, University at Buffalo 12292 , Buffalo, New York, USA; Samsung Medical Center, Seoul, Republic of Korea

**Keywords:** new β-lactam/β-lactamase inhibitors, PBP3-mediated resistance, *Escherichia coli*

## Abstract

*Escherichia coli* with penicillin-binding protein 3 (PBP3) insertions appeared in the early 2000s and expanded during the 2010s. The YRIN and YRIK variants predominate globally, while other insertion types remain rare. These insertions frequently co-occur with carbapenemases (NDM-5 and OXA-181) and high-risk clones (ST410, ST167, and ST405), contributing to the evolution and dissemination of multidrug resistance. Newer β-lactam/β-lactamase inhibitor combinations (ceftazidime/avibactam, aztreonam/avibactam, and cefepime/taniborbactam) and cefiderocol with affinities for PBP3 show reduced activity against *E. coli* with YRIN and YRIK insertions. The continued spread of such strains threatens the effectiveness of these β-lactam agents. In contrast, agents targeting PBP2, such as cefepime/zidebactam and cefiderocol-xeruborbactam, retain potent *in vitro* activity and will likely be important for future treatment strategies. Laboratory methods to detect PBP3 insertions are lacking, raising concern that these strains may be spreading largely undetected. Current surveillance efforts are insufficient, leaving significant gaps in data describing the global distribution and prevalence of *E. coli* with PBP3 insertions. The convergence of β-lactamase production and PBP target modification represents a paradigm shift within *E. coli* toward multidrug resistance. This occurrence provides a major barrier to current and future β-lactam/β-lactamase inhibitor development. There is an urgent need for enhanced global surveillance, along with rapid diagnostic tools capable of identifying PBP3 insertions and associated high-risk clones among carbapenemase-producing *E. coli*. Future strategies should prioritize agents with activities against multiple essential PBPs to preserve β-lactam efficacy.

## INTRODUCTION

Antimicrobial resistance (AMR) is one of the largest menaces to human health (1). In 2019, an estimated 4.95 million deaths worldwide were associated with bacterial AMR, including 1.27 million deaths attributable to bacterial AMR (2). There is a scarcity of effective antibiotics for treating multidrug-resistant (MDR) gram-negative bacterial infections (3).

*Escherichia coli* are divided into three major pathotypes: (i) commensal organisms residing in the gastrointestinal tract; (ii) enteric pathogens that cause intestinal disease; and (iii) extraintestinal pathogenic *E. coli* (ExPEC) that cause infections beyond the gastrointestinal tract (4). Throughout this article, *E. coli* denote ExPEC unless otherwise specified.

*E. coli* is the most frequent cause of community-associated urinary and bloodstream infections worldwide (5). Bloodstream infections caused by MDR *E. coli* are linked with high mortalities, extended hospital stays, and increased health care expenditures (6). During 2019, MDR *E. coli* caused just over 800,000 global deaths (2).

Before the 2000s, *E. coli* isolates causing human infections were mainly susceptible to a variety of antimicrobial agents (7). During the 2000s and 2010s, MDR *E. coli* increased globally (8). This worldwide increase in MDR *E. coli* led to the overutilization of carbapenems with subsequent carbapenem resistance (9).

This review article is aimed at the “non-specialist” to provide an overview of the mechanisms of β-lactam resistance among *E. coli,* MDR high-risk *E. coli* clones, and newer β-lactam/β-lactamase inhibitor combinations. We also describe *E. coli* penicillin-binding protein (PBP) affinities and modifications, and the potential implications of PBP3 insertions for newer β-lactam/β-lactamase inhibitor combinations.

## β-LACTAM RESISTANCE AMONG *E. COLI*

β-Lactam drugs are often used to treat infections due to *E. coli* (7, 10, 11). The efficacy of β-lactam agents mainly depends on three factors (12): (i) penetrating the gram-negative outer membrane via protein channels; (ii) binding to and inactivating their targets called PBPs; and (iii) the avoidance of enzymes (called β-lactamases) that can inactivate them. The production of β-lactamases and PBP modifications are important causes of β-lactam resistance among *E. coli* (7).

### β-Lactamases among *Escherichia coli*

β-Lactamases are enzymes that hydrolyze the β-lactams, resulting in ineffectual compounds (13), and are divided into four classes (i.e., A, B, C, and D) (14). Classes A, C, and D comprise serine β-lactamases, in which a serine residue is essential for catalytic activity, whereas class B enzymes are metallo-β-lactamases (MBLs) that require zinc ions at their active site (13). Carbapenem-hydrolyzing β-lactamases are found predominantly within class A (e.g., KPC enzymes), class B (e.g., NDM, VIM, and IMP enzymes), and class D (e.g., OXA-type carbapenemases) (15, 16).

The production of β-lactamases constitutes the principal mechanism by which *E. coli* develops resistance to β-lactam agents (17) and belong to three groups, namely: (i) extended-spectrum β-lactamases (ESBLs), consisting most often of the CTX-M types; (ii) AmpC β-lactamases; and (iii) carbapenemases (18). Among carbapenemase-producing *E. coli*, OXA-48-like, NDM, and KPC enzymes are encountered most frequently, whereas IMP and VIM carbapenemases remain comparatively uncommon (19). *E. coli* harboring NDM or OXA-48-like carbapenemases have been reported outside healthcare settings, highlighting their establishment within community populations (20, 21).

### Multidrug-resistant *Escherichia coli* clones

The dissemination of AMR determinants among *E. coli* populations occurs through two processes (22): (i) globally distributed MDR lineages, often referred to as high-risk clones, have acquired AMR genes and (ii) mobile genetic elements (e.g., plasmids).

Specific AMR determinants are frequently linked to certain *E. coli* clonal backgrounds that include the following (23): (i) ST131, ST405, ST38, ST648, ST410, ST167, and ST1193 with fluoroquinolone resistance; (ii) ST131, ST405, ST38, and ST648 with CTX-M β-lactamases; and (iii) ST131, ST410, ST405, and ST167 with carbapenemases (23, 24).

### *Escherichia coli* penicillin-binding proteins

Cell wall synthesis consists of three stages, namely the cytoplasmic or stage 1, the membrane or stage 2, and the periplasmic or stage 3 (25). The third stage consists of glycan chain polymerization and the cross-linking of peptide side chains within the peptidoglycan layer (25). PBPs are transpeptidases or carboxypeptidases that catalyze the late steps, including glycan polymerization and cross-linking of peptide side chains (26). β-Lactam drugs bind to and inactivate PBPs, causing bacterial death through weakening of the peptidoglycan layer (27).

Penicillin-binding proteins in *E. coli* are divided into two main groups (28): (i) high-molecular-weight or essential PBPs and (ii) low-molecular-weight or non-essential PBPs. High-molecular-weight PBPs consist of two classes: class A includes PBP1a and PBP1b, while class B consists of PBP2 and PBP3. High-molecular-weight PBPs are essential for the viability, growth, and division of bacteria. The low-molecular-weight PBPs consist of a single class named class C, which includes PBP4 to PBP8. Low-molecular-weight PBPs are responsible for shaping the cell wall during growth, division, and adaptation to stress. PBPs are unique to bacteria and are excellent antibiotic targets (29).

### Affinity of β-lactams to *E. coli* penicillin-binding proteins

Affinity is the process of β-lactam binding to PBPs and subsequent inactivation (27–29). This complex process includes the following principles: (i) β-lactams that target and inactivate essential PBPs will produce rapid bacterial death with high population elimination; (ii) β-lactams that mainly target non-essential PBPs will produce slower bacterial death, resulting in less population elimination; (iii) β-lactams that inactivate multiple essential and non-essential PBPs will produce more rapid bacterial death (compared to agents that only target a single PBP), resulting in even higher population extinction; and (iv) the modification of PBP structures can lead to less effective β-lactam-PBP binding, resulting in decreased affinity with the subsequent survival of bacteria.

The affinity of β-lactams for PBPs is assessed using biochemical, biophysical, microbiological, and structural methods (27, 30). Radiolabeled β-lactam-binding assays that determine the direct binding and acylation of PBPs by β-lactams are considered the classical gold standard method (27). Minimum inhibitory concentration (MIC) increases among isogenic strains when comparing wild-type PBPs to those with modified PBPs, serving as a biological method that indirectly indicates reduced binding affinity (31).

The affinities of ceftazidime, cefepime, aztreonam, meropenem, and imipenem to different *E. coli* PBPs and genes encoding each PBP are displayed in Table 1 (27, 30). These agents were chosen because they are used in combination with newer β-lactamase inhibitors. Cefiderocol was included because its affinity for multiple PBPs included PBP3 (32). In summary, the primary PBP target of ceftazidime, cefepime, aztreonam, and cefiderocol is PBP3 (encoded by *ftsI*), while the primary target of meropenem and imipenem is PBP2 (encoded by *mrdA*). Note that ceftazidime, cefepime, meropenem, and imipenem also inactivate other essential PBPs, while meropenem and imipenem also target non-essential PBPs (Table 1).

**TABLE 1:** β-Lactam affinities for *Escherichia coli* PBPs^*a*^

β-Lactam	PBP1a (*ponA*)	PBP1b (*ponB*)	PBP2 (*mrdA*)	PBP3 (*ftsI*)	PBP4 (*dacB*)	PBP5/6 (*dacA*)	PBP7 (*pbpG*)	PBP8 (*pbpH*)
Ceftazidime	2+	2+	1+	3+	N	N	N	N
Cefepime	1+	1+	1+	3+	N	N	N	N
Aztreonam	N	N	N	3+	N	N	N	N
Meropenem	N	N	3+	2+	3+	N	1+	1+
Imipenem	2+	2+	3+	1+	3+	1+	N	N
Cefiderocol	1+	1+	1+	3+	N	N	N	N

^*a*^ Genes responsible for encoding PBPs are given in brackets. Essential PBPs: class A includes 1a, 1b, and class B includes 2, 3. Non-essential PBPs: class C includes 4, 5/6, 7, 8. Affinity: 3+, high (primary PBP target); 2+, moderate (secondary PBP target); 1+, low affinity (tertiary PBP target); N, no/neglectable/unknown. Affinity based on data from references 27 and 30.

## *E. COLI* PBP3 INSERTIONS

### The initial report: YRIN and YRIK

**Fig 1:**
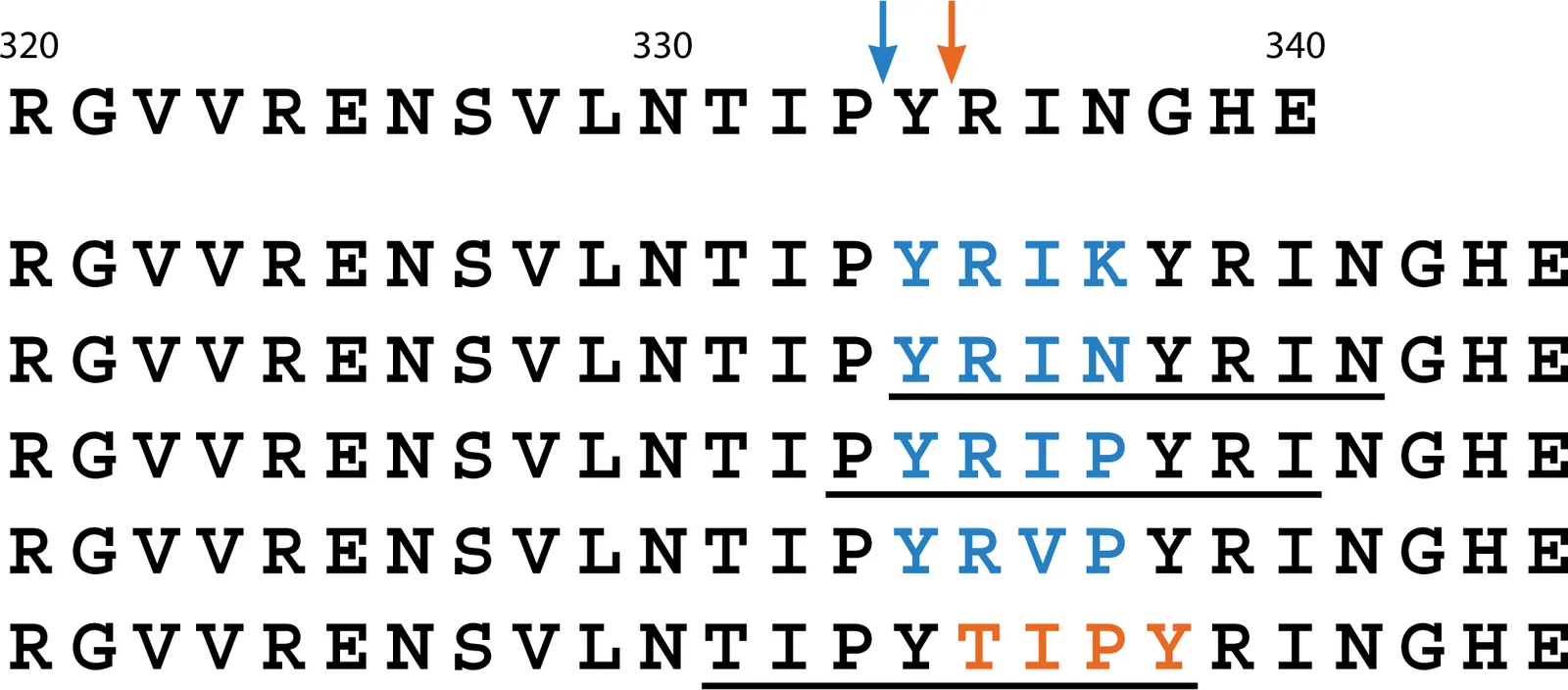
Different *Escherichia coli* PBP3 insertions. Top: wild-type PBP three from amino acid (aa) positions 320 to 340. Blue arrow: insertions (YRIK, YRIN, YRIP, and YRVP) between aa 333 and 334. Green arrow: insertion (TIPY) between aa 334 and 335. Duplications (YRIN_YRIN, PYRI_PYRI, and TIPY_TIPY) are underlined. Y, tyrosine; R, arginine; I, isoleucine; N, asparagine; K, lysine; P, proline; T, threonine; V, valine.

The AstraZeneca surveillance program from the early 2010s identified 14 NDM-producing *E. coli* isolates with high MICs to aztreonam/avibactam, ranging from 4/4 to 16/4 µg/mL (31). Whole-genome sequencing (WGS) showed the insertion of four amino acids within PBP3 after amino acid (aa) position 333 that were named YRIK and YRIN (Fig. 1). The YRIN insertion led to the YRIN_YRIN duplication at aa positions 334–341 (Fig. 1). The *ftsI* that contained YRIK was cloned into *E. coli* ATCC 25922 (31). The MICs to ceftriaxone, cefepime, and aztreonam increased significantly (from 0.03 to 4 µg/mL), while the MICs to meropenem and imipenem remained the same. The MIC increases were attributed to the YRIK insertion and were likely due to decreased affinity of ceftriaxone, cefepime, and aztreonam to PBP3. Structural analysis was based on molecular modeling and suggested that both insertions occurred in the β2b–β2c loop, which may have impacted the accessibility of the β-lactam drugs to the transpeptidase pocket of PBP3 (31). A genomic *E. coli* ST410 study from 2022 reported the YRIK insertion as KYRI (33).

### Other PBP3 insertions: TIPY, YRIP, and TRVP

Investigators from the USA obtained a clinical *E. coli* in 2017 with *bla*_KPC-2_ and ceftazidime/avibactam and aztreonam/avibactam MICs of 8/4 µg/mL to both drugs (34). WGS identified a different four amino acid PBP3 insertion after aa position 334 that was named TIPY, resulting in a TIPY_TIPY duplication at aa positions 331–341 (Fig. 1). The location of the TIPY insertion is in a loop adjacent to the PBP3 active site and likely prevents the entry of β-lactam agents (34).

Chinese investigators collected *E. coli* with *bla*_NDM-1_ a year later from a patient’s urine with aztreonam/avibactam MIC of 8/4 µg/mL (35). WGS identified a YRIP insertion after aa position 333 that led to PYRI_PYRI duplication at aa positions 333–340 (Fig. 1).

In 2023, Swiss investigators obtained a broad-spectrum cephalosporin- and aztreonam-resistant clinical *E. coli* that tested negative for ESBLs and AmpC β-lactamases (36). The ceftazidime/avibactam MIC was 8/4 µg/mL. WGS identified a YRVP insertion after position 333 (Fig. 1). Wild-type PBP3 was cloned and transformed into the clinical isolate, and cephalosporin and aztreonam sensitivities were restored, suggesting that this insertion was responsible for the increased MICs to the broad-spectrum cephalosporins, aztreonam, and ceftazidime/avibactam (36).

Rare PBP3 duplications (e.g., TVVP, TVPYTVPY, TVPYPVPY, VGDR, and ANALNIPL) were reported in a bioinformatic-based study (37). However, it is unclear if these duplications resulted in increased MICs to newer β-lactam/β-lactamase inhibitor combinations.

### PBP3 insertions: global frequencies

The overall global prevalence and distribution of *E. coli* with PBP3 insertions are difficult to ascertain because published genomic surveillance studies are scarce and used different inclusion criteria when reporting on PBP3 insertions. Inclusion criteria included the following: no selection criteria (37), non-susceptibility to ceftazidime/avibactam (38), or aztreonam/avibactam (39) and/or cefiderocol (40), and carbapenemase-producing isolates (41, 42).

A large-scale bioinformatic-based study downloaded all *E. coli* genomes (as of November 2023) from Enterobase (37). Among 159,341 high-quality *E. coli* genomes, investigators found that 2% contained PBP3 insertions, with the YRIN duplication being the most frequent at 66%, followed by YRIK (e.g., 29%), and the PYRI duplication at 4%. The TIPY duplication and the YRVP insertion were rare. Genomes that contained YRIN, YRIK, and PYRI were submitted from 62 countries, most often from the USA, China, Germany, Australia, and Canada, reflecting geographic locations in the ability to perform WGS. The interesting aspect from that study was that the first YRIN duplication-containing genome was from 2002, while the first YRIK-containing genome was from 2010, and the first PYRI duplication occurred in 2012 (37).

The SIDERO-WT-2014 surveillance study (from Europe and North America) found 65/1,529 *E. coli* isolates with MICs ≥ 2 µg/mL for ceftazidime/avibactam, and/or ceftolozane/tazobactam, and/or cefiderocol (38). Eleven of these *E. coli* were obtained from Turkey, USA, and Italy and possessed the YRIN duplication.

A surveillance study that consisted of 64 medical centers from Europe, the Asia-Pacific region, and Latin America included 8,787 Enterobacterales collected consecutively in 2019 (39). Within this collection, 11 *E. coli* isolates (obtained from Turkey, Korea, Vietnam, Thailand, France, Italy, and Australia) showed an aztreonam/avibactam MIC of ≥4/4 mg/L; nine isolates contained the YRIK insertion, and two isolates contained the YRIN duplication.

During 2020 and 2025, the Maryland Department of Health Laboratories Administration in the USA received 40 clinical isolates of NDM-producing *E. coli* from surrounding laboratories; 18 (45%) were positive for the YRIN duplication, and 12 (30%) for the YRIK insertion (41).

Imported cases of NDM-5-producing *E. coli* ST167 and ST361 with YRIN duplications have been reported from the USA (imported from India) (43) and from Germany (imported from Ukraine) (40).

### PBP3 insertions: associations with β-lactamases and high-risk clones

It became apparent early on that strains containing PBP3 insertions (specifically YRIN and YRIK) are associated with *bla*_NDMs_, *bla*_OXA-48-like_, *bla*_CTX-M_, and plasmid-mediated AmpC β-lactamases. The most common individual β-lactamases linked with these strains included the following: NDM-5 (37), OXA-181 (37), CTX-M-15 (44), and CMY-42 (45). Overall, PBP3 insertions are scarce among *E. coli* with *bla*_KPCs_, *bla*_VIMs_, and *bla*_IMPs_.

There are interesting associations of strains containing YRIN, YRIK, and PYRI with certain *E. coli* high-risk clones (37). YRIN is frequent among the following high-risk clones (e.g., ST410, ST167, ST648, ST617, ST361, ST101, ST1284, and ST448); YRIK is frequent among ST410, ST405, ST648, ST617, and ST38, while PYRI occurs among ST410 and ST354 (37, 42). Among global carbapenemase-producing *E. coli,* the prevalence of PBP3 insertions among high-risk clones was as follows: 75% of ST410 and 100% of ST167 contained YRIN, while 10% of ST410 and 50% of ST405 contained YRIK (42).

**Fig 2:**
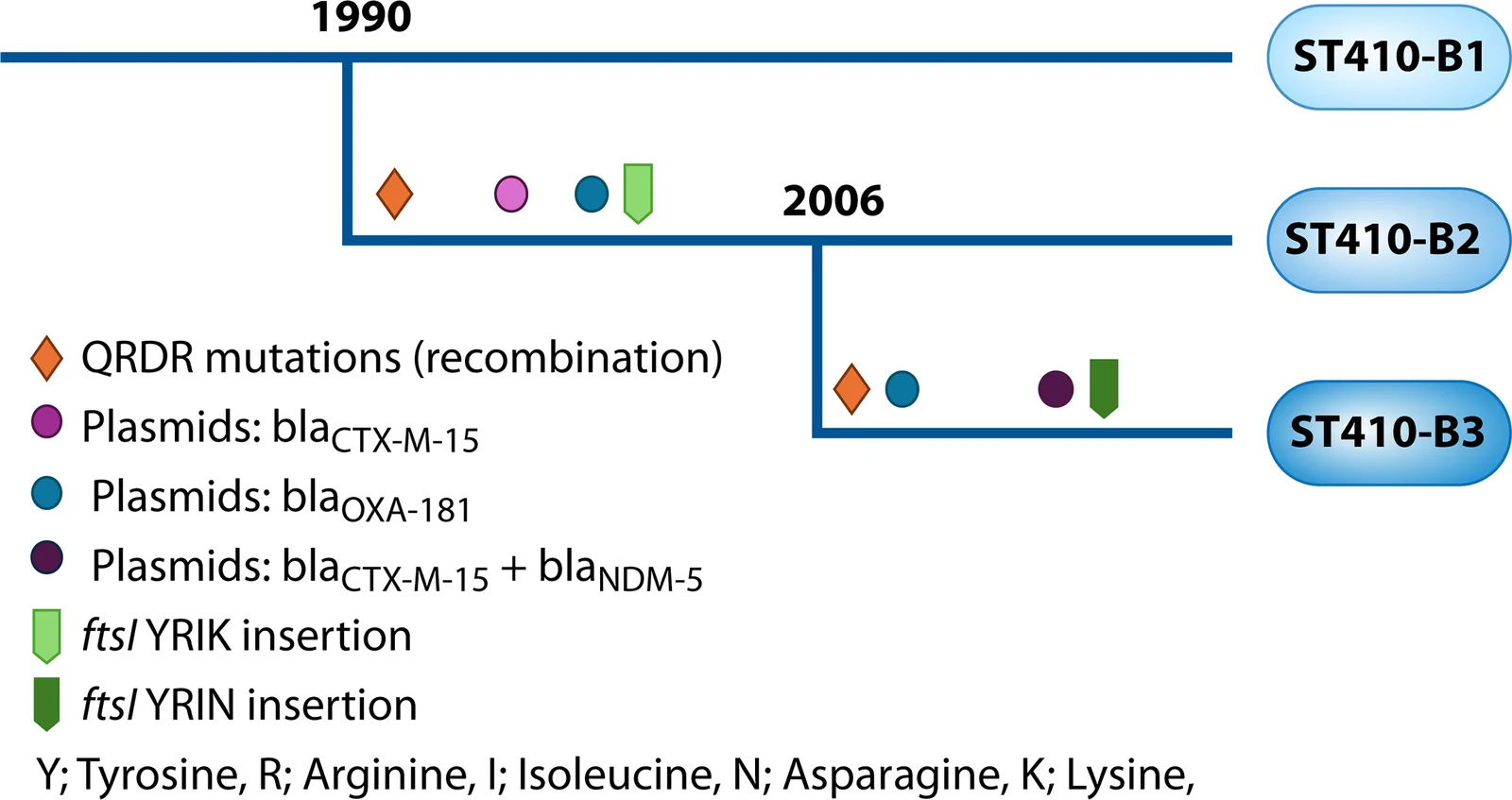
The acquisition over time of YRIK and YRIN PBP3 insertions in ST410 subclades.

The acquisition of PBP3 insertions has played an important role in the evolution of ST410 (33, 46). ST410 was responsible for the global distribution of OXA-181 in the early to mid-2010s (21). In the late 2010s, this clone had also acquired NDM-5 (33). ST410 belongs to two clades, namely A and B. Clade B is divided into three subclades, namely B1, B2, and B3 (Fig. 2) (46). YRIK is found in some (approximately 20%) of ST410-B2, while YRIN is present among most ST410-B3 (approximately 80%). Characterization of the regions surrounding the *ftsl* gene showed that the ST410 region is very similar to the *ftsl* gene found in ST167 (33). When ST410-B2 evolved into ST410-B3, the YRIK insertion was replaced by YRIN due to the change of lysine to asparagine (Fig. 2). The YRIN duplication then became established among the B3 subclade that dominates the population structure of this lineage (46). The ST410-B3 subclade has shown a stepwise progression towards developing MDR (Fig. 2).

### PBP3 insertions: laboratory detection

The laboratory detection of *E. coli* strains harboring PBP3 insertions remains in the early stages of development. A pentaplex PCR assay has been described that enables the simultaneous detection of *bla*_CTX-M_, *bla*_OXA-1_, *bla*_CMY_, *bla*_NDM_, and the common PBP3 insertion variants (YRIN and YRIK) in a single reaction (47).

The presence of *bla*_CMY_ in combination with *bla*_CTX-M_ in NDM-producing *E. coli* carrying YRIN or YRIK insertions is associated with elevated aztreonam–avibactam MICs (>4 µg/mL) (44, 45). Isolates without *bla*_CMY_ often test sensitive to aztreonam–avibactam(<4 µg/mL) and remain difficult for clinical laboratories to detect (48).

### PBP3 insertions: summary

The characteristics of *E. coli* with PBP3 insertions are summarized in Table 2. *E. coli* with PBP3 insertions first appeared in the early 2000s and have increased in numbers during the late 2010s. The three most common global types are the YRIN, YRIK, and PYRI. The prevalence of strains with these PBP3 insertions is difficult to ascertain due to different selection criteria used in surveillance studies. Surveillance data (suboptimal at best) do suggest that isolates with YRIN and YRIK insertions have a global distribution and that a significant number of global isolates (close to 50%) with *bla*_NDMs_ also contain PBP3 insertions. Certain β-lactamases (e.g., NDM-5, OXA-181, CTX-M-15, and CMY-42) are associated with *E. coli* with YRIN and YRIK insertions. Strains with *bla*_CMY-42_ showed high aztreonam/avibactam MICs. There is a large clonal component among *E. coli* with PBP3 insertions: YRIN is linked with ST410-B3, ST167, ST648, and ST361, whereas YRIK is linked with ST405 and ST410-B2. Clonal data about PYRI are scarce and have been found in ST410 (unknown subclade) and ST354.

**TABLE 2:** Characteristics of *Escherichia coli* with PBP3 insertions^*a*^

PBP3 insertion	Distribution	Occurrence^*c*^	β-Lactamases^*b*^	High-risk clones^*b*^
YRIN	Global (first described in India)	Most frequent	NDM-5, OXA-181, CMY-42, CTX-M-15	ST410-B3, ST167, ST648, ST361
YRIK	Global (first described in India)	Second most frequent	NDM-5, OXA-181, CMY-42, CTX-M-15	ST405, ST410-B2
PYRI	Unknown (first described in China)	Less often	Not known	ST410, ST354
TIPY	Unknown (first described in USA)	Rare	Not known	Not known
TRVP	Unknown (first described in Switzerland)	Rare	Not known	Not known

^*a*^ Y, tyrosine; R, arginine; I, isoleucine; N, asparagine; K, lysine; P, proline; T, threonine; V, valine.

^*b*^ Most frequent β-lactamases and high-risk clones among *E. coli* with PBP3 insertions.

^*c*^ Insufficient data due to different inclusion criteria of surveillance studies.

## NEWER β-LACTAM/β-LACTAMASE INHIBITOR COMBINATIONS

### Introduction

The idea to restore the activity of β-lactam agents by combining them with β-lactam inhibitors originated during the 1960s and 1970s (10). β-Lactamase inhibitor binds to the β-lactamase and inactivates the enzyme. This enables the β-lactam agent partner to inactivate the PBP target, resulting in bacterial cell death (49).

β-Lactamase inhibitors are divided into two groups: the “older” or suicide group and the “newer” or non-suicide inhibitors (49, 50). Suicide inhibitors bind covalently to β-lactamases in an irreversible fashion (10). These inhibitors (e.g., clavulanate, sulbactam, and tazobactam) are structurally related to β-lactam antibiotics. Non-suicide inhibitors bind non-covalently to β-lactamases in a reversible fashion (50). These inhibitors (e.g., avibactam, relebactam, vaborbactam, etc.) are not structurally related to β-lactam antibiotics.

We provide some general concepts regarding newer inhibitors and β-lactam/β-lactamase inhibitor combinations (49–52): (i) newer β-lactamase inhibitors (e.g., nacubactam, ledaborbactam, funobactam, etc.) and cefiderocol/xeruborbactam are currently in phase 1 or early phase 3 trials, will not be discussed in detail. (ii) Newer β-lactam/β-lactamase inhibitor combinations were designed to treat infections with carbapenemase-producing bacteria. (iii) Most of the newer β-lactamase inhibitors (e.g., avibactam, relebactam, zidebactam, vaborbactam, and taniborbactam) inhibit class A enzymes (including KPCs and ESBLs), as well as class C enzymes (e.g., AmpC) (Table 3). (iv) Some newer β-lactamase inhibitors (e.g., avibactam, zidebactam, taniborbactam, and durlobactam) will inhibit class D enzymes (e.g., OXA-48-like carbapenemases) (Table 3). (v) Limited number of inhibitors (e.g., taniborbactam and xeruborbactam) will inhibit the MBLs (Table 3). (vi) Taniborbactam does not inhibit the IMP types of MBLs. (vii) Some newer inhibitors are called β-lactam enhancers (e.g., zidebactam and durlobactam) and do more than just β-lactamase inhibition (e.g., binding to PBP2). (viii) The following β-lactam agents are combined with newer inhibitors: cefepime, ceftazidime, cefiderocol, aztreonam, meropenem, and imipenem (Table 3). (ix) Aztreonam has been used in β-lactam/β-lactamase inhibitor combinations because of its activity against MBLs. (x) Newer β-lactamase inhibitors consist of two structural types: diazabicyclo-octane-based inhibitors and the boronic acid based inhibitors.

**TABLE 3:** Newer β-lactam/β-lactamase inhibitor combinations: inhibitions and activities^*c*^

β-Lactamase inhibitor	β-Lactamase inhibition^*b*^	β-Lactam	β-Lactam/β-lactamase inhibitor combination: activities
Avibactam	Classes A, C, D	Ceftazidime	KPCs^*a*^, OXA-48-like, ESBLs, AmpCs
		Aztreonam	KPCs^*a*^, MBLs, OXA-48-like, ESBLs, AmpCs
Relebactam	Classes A, C	Imipenem	KPCs^*a*^, ESBLs, AmpCs
Durlobactam	Classes A, C, D	Sulbactam	OXA-carb
Zidebactam	Classes A, C, D	Cefepime	KPCs^*a*^, MBLs, OXA-carb, ESBLs, AmpCs
Vaborbactam	Classes A, C	Meropenem	KPCs^*a*^, ESBLs, AmpCs
Taniborbactam	Classes A, B, C, D	Cefepime	KPCs^*a*^, MBLs (not IMPs), OXA-carb, ESBLs, AmpCs
Xeruborbactam	Classes A, B, C, D	Cefiderocol	KPCs^*a*^, MBLs, OXA-carb, ESBLs, AmpCs

^*a*^ Include other class A carbapenemases (such as GES, IMI, and SME).

^*b*^ Ambler classes are based on amino acid sequence similarities.

^*c*^ ESBLs, extended-spectrum β-lactamases; MBLs, metallo-β-lactamases; OXA-carb, OXA carbapenemases.

### Diazabicyclo-octane-based β-lactamase inhibitors

Diazabicyclo-octane-based inhibitors are divided into two subgroups, namely those that are β-lactamase inhibitors and those inhibitors that are also β-lactamase enhancers.

#### β-Lactamase inhibitors

Avibactam is a class A, C, and some D β-lactamase inhibitor (53) (Table 3). It is combined with ceftazidime, which was released in 2015 for complicated intra-abdominal infections (IAIs), complicated urinary tract infections (UTIs), and hospital-acquired pneumonia (HAP) (54). This compound has activity against bacteria with KPC carbapenemases, other class A carbapenemases (such as GES, IMI, and SME), the OXA-48-like carbapenemases, ESBLs, and AmpCs (53, 54) (Table 3). A more recent combination is with aztreonam that has been available since 2024 in Europe for complicated IAIs, UTIs, and HAP (55). This combination received FDA approval in 2025 for complicated intra-abdominal infections (56). Aztreonam/avibactam has activity against bacteria with KPC carbapenemases, other class A carbapenemases (such as GES, IMI, and SME), the OXA-48-like carbapenemases, ESBLs, AmpCs, and MBLs (due to the aztreonam) (Table 3) (55).

Relebactam is a class A and C inhibitor (57) and, combined with imipenem, was released in 2019 for complicated IAIs, UTIs, and HAP (58) (Table 3). Imipenem/relebactam has activity against KPC carbapenemases, other class A carbapenemases (such as GES, IMI, and SME), ESBLs, and AmpCs (Table 3) (57).

#### β-Lactamase enhancers

Zidebactam is a class A, C, and D inhibitor and is combined with cefepime (58) (Table 3). That compound has completed a phase 3 trial for complicated UTIs and had recently been approved at the FDA (52). Cefepime/zidebactam has activity against bacteria with various β-lactamases including MBLs (Table 3) (52). The MBL activity is likely due to the affinity of zidebactam for PBP2, although the exact mechanism is unclear (59).

Durlobactam is a class A, C, and D inhibitor and enhancer, that is combined with sulbactam and was released in 2023 in the USA for HAP specifically due to OXA carbapenemase-producing *Acinetobacter baumannii* (Table 3) (60).

### Boronic acid-based inhibitors

Vaborbactam is a class A and C inhibitor and, combined with meropenem, was released in 2017 (USA) and 2018 (Europe) for complicated UTIs (61) (Table 3). This compound has activity against KPC carbapenemases, other class A carbapenemases (such as GES, IMI, and SME), ESBLs, and AmpCs (62) (Table 3).

Taniborbactam is a class A, B, C, and D inhibitor and is combined with cefepime (63). This compound has completed a phase 3 trial for complicated UTIs (64). Cefepime/taniborbactam has activity against KPCs, MBLs (not IMPs), and OXA carbapenemases (Table 3), and it is unclear if this compound will make it to the market (65).

Xeruborbactam is a class A, B, C, and D inhibitor, combined with cefiderocol, and is currently in late phase 3 trials (52). Cefiderocol/xeruborbactam has activity against KPCs, MBLs, and OXA carbapenemases (Table 3) (66).

### Newer β-lactam/β-lactamase inhibitor combinations: summary

Newer β-lactam/β-lactamase inhibitor combinations have excellent activity against class A carbapenemases and good activity against class D carbapenemases (56) (67)(Table 3). Unfortunately, inhibition of class B or MBLs remains problematic (50). At present, taniborbactam is the only β-lactamase inhibitor in advanced development that inhibits MBLs (52). Xeruborbactam has activity against most IMPs and NDM variants but is still in late phase 3 clinical trials (52). The β-lactam enhancer, zidebactam, does not actually inhibit class B β-lactamases, but in combination with cefepime, has shown excellent activities against MBL-producing organisms (68).

## *ESCHERICHIA COLI* WITH PBP3 INSERTIONS: IMPLICATION FOR NEWER β-LACTAM/β-LACTAMASE INHIBITOR COMBINATIONS

*E. coli* with PBP3 insertions (specifically with YRIN and YRIK) are found mainly among strains that also contain *bla*_NDMs_ (Table 2) (37). Newer β-lactam/β-lactamase inhibitor combinations with activities against MBL-producing *E. coli* include aztreonam/avibactam, cefepime/zidebactam, cefepime/taniborbactam, and meropenem/xeruborbactam (51, 52). The activities of cefepime/zidebactam and cefepime/taniborbactam were compared against NDM-producing *E. coli* with YRIN and YRIK insertions, showing high aztreonam/avibactam MICs (ranging from 1/4 to 16/4 µg/mL) (69). Cefepime/taniborbactam had limited or no activity against these strains, while cefepime/zidebactam showed very low MICs (≤0.125 µg/mL). A different study compared the activities of cefepime/taniborbactam and meropenem/xeruborbactam against *E. coli* with YRIN and YRIK insertions, with high aztreonam/avibactam MICs (ranging from 1/4 to >64/4 µg/mL) (70). Cefepime/taniborbactam again had limited or no activity, while meropenem/xeruborbactam showed very low MICs (≤0.25 µg/mL) against such strains.

Data on strains co-possessing PBP3 insertions and *bla*_OXA-181_ are still rare. Therefore, the authors will not describe the details of the activities of newer β-lactam/β-lactamase inhibitor combinations against strains with *bla*_OXA-181_ but rather focus on *bla*_NDMs_. However, ceftazidime/avibactam is often used to treat infections with *E. coli* that produce OXA-48-like carbapenemases (58). *E. coli* with PBP3 insertions have shown increased MICs to ceftazidime/avibactam but do not always test as non-susceptible using EUCAST breakpoints (71). It will be important to monitor for increases in prevalence of PBP3 insertions among *E. coli* that produce OXA-48-like carbapenemases.

In summary, aztreonam/avibactam, cefepime/zidebactam, and cefepime/taniborbactam have good affinities for PBP3 (due to the aztreonam and cefepime) and showed limited activities against strains with PBP3 insertions. Cefepime/zidebactam has good affinities for PBP2 (due to zidebactam) and showed excellent *in vitro* activities against strains with PBP3 insertions.

### Conclusions

The emergence of *E. coli* with PBP3 insertions represents a convergence of AMR mechanisms, combining β-lactamase production (e.g., NDM) with target-site modification (e.g., PBP3 insertions). Strains with YRIN and YRIK PBP3 insertions are associated with high-risk clones (e.g., ST410, ST167, and ST405) and show reduced susceptibility to several newer β-lactam/β-lactamase inhibitor combinations (e.g., ceftazidime/avibactam, aztreonam/avibactam, and cefepime/taniborbactam) as well as cefiderocol. In contrast, agents with strong PBP2 affinity appear to retain activity, highlighting the importance of multi-PBP targeting strategies.

A major challenge is the lack of methods to detect such strains in clinical practice. Isolates harboring YRIN and YRIK may test sensitive to some newer β-lactam/β-lactamase inhibitor combinations, masking their presence and leading to silent dissemination. Current diagnostic capabilities for detecting *E. coli* with PBP3 insertions and identifying associated high-risk clones remain limited, and approaches suitable for routine laboratories are urgently needed.

Enhanced global surveillance and improved diagnostics will be essential to define the epidemiology and limit the spread of these *E. coli* strains. Future antimicrobial development should prioritize agents with activity against multiple essential PBPs to mitigate the impact of single-target resistance mechanisms. Together, these efforts will be critical to preserving the effectiveness of β-lactam therapies against increasingly complex MDR *E. coli*. The emergence and spread of *E. coli* with *bla*_NDMs_ harboring PBP3 insertions underscore the limitation of therapeutic approaches focused solely on β-lactamase inhibition.

## References

[1] Baquero F. 2021. Threats of antibiotic resistance: an obliged reappraisal. Int Microbiol 24:499–506. doi:10.1007/s10123-021-00184-y PMC8141826 34028624

[2] Antimicrobial Resistance C. 2022. Global burden of bacterial antimicrobial resistance in 2019: a systematic analysis. Lancet 399:629–655. 10.1016/S0140-6736(21)02724-0 PMC8841637 35065702

[3] Tacconelli E, Carrara E, Savoldi A, Harbarth S, Mendelson M, Monnet DL, Pulcini C, Kahlmeter G, Kluytmans J, Carmeli Y, Ouellette M, Outterson K, Patel J, Cavaleri M, Cox EM, Houchens CR, Grayson ML, Hansen P, Singh N, Theuretzbacher U, Magrini N, WHO Pathogens Priority List Working Group. 2018. Discovery, research, and development of new antibiotics: the WHO priority list of antibiotic-resistant bacteria and tuberculosis. Lancet Infect Dis 18:318–327. doi:10.1016/S1473-3099(17)30753-3 29276051

[4] Pitout JDD. 2012. Extraintestinal pathogenic *Escherichia coli*: a combination of virulence with antibiotic resistance. Front Microbiol 3:9. doi:10.3389/fmicb.2012.00009 PMC3261549 22294983

[5] Laupland KB, Church DL. 2014. Population-based epidemiology and microbiology of community-onset bloodstream infections. Clin Microbiol Rev 27:647–664. doi:10.1128/CMR.00002-14 PMC4187633 25278570

[6] Bonten M, Johnson JR, van den Biggelaar AHJ, Georgalis L, Geurtsen J, de Palacios PI, Gravenstein S, Verstraeten T, Hermans P, Poolman JT. 2021. Epidemiology of *Escherichia coli* bacteremia: a systematic literature review. Clin Infect Dis 72:1211–1219. doi:10.1093/cid/ciaa210 32406495

[7] Pitout JDD. 2012. Extraintestinal pathogenic *Escherichia coli*: an update on antimicrobial resistance, laboratory diagnosis and treatment. Expert Rev Anti Infect Ther 10:1165–1176. doi:10.1586/eri.12.110 23199402

[8] Peirano G, Endimiani A, Pitout JDD. 2025. CTX-M-producing *Escherichia coli*: history, molecular epidemiology and laboratory detection. Infect Drug Resist 18:6549–6560. doi:10.2147/IDR.S553853 PMC12702286 41399812

[9] Tompkins K, van Duin D. 2021. Treatment for carbapenem-resistant enterobacterales infections: recent advances and future directions. Eur J Clin Microbiol Infect Dis 40:2053–2068. doi:10.1007/s10096-021-04296-1 PMC8527571 34169446

[10] Drawz SM, Bonomo RA. 2010. Three decades of beta-lactamase inhibitors. Clin Microbiol Rev 23:160–201. doi:10.1128/CMR.00037-09 PMC2806661 20065329

[11] Papp-Wallace KM, Endimiani A, Taracila MA, Bonomo RA. 2011. Carbapenems: past, present, and future. Antimicrob Agents Chemother 55:4943–4960. doi:10.1128/AAC.00296-11 PMC3195018 21859938

[12] Pitout JD, Sanders CC, Sanders WE Jr. 1997. Antimicrobial resistance with focus on beta-lactam resistance in gram-negative bacilli. Am J Med 103:51–59. doi:10.1016/s0002-9343(97)00044-2 9236486

[13] Bonomo RA. 2017. β-lactamases: a focus on current challenges. Cold Spring Harb Perspect Med 7:a025239. doi:10.1101/cshperspect.a025239 PMC5204326 27742735

[14] Ambler RP, Coulson AF, Frère JM, Ghuysen JM, Joris B, Forsman M, Levesque RC, Tiraby G, Waley SG. 1991. A standard numbering scheme for the class a beta-lactamases. Biochem J 276 ( Pt 1):269–270. doi:10.1042/bj2760269 PMC1151176 2039479

[15] Pitout JD, Nordmann P, Poirel L. 2015. Carbapenemase-producing *Klebsiella pneumoniae,* a key pathogen set for global nosocomial dominance. Antimicrob Agents Chemother 59:5873–5884. doi:10.1128/AAC.01019-15 PMC4576115 26169401

[16] Poirel L, Pitout JD, Nordmann P. 2007. Carbapenemases: molecular diversity and clinical consequences. Future Microbiol 2:501–512. doi:10.2217/17460913.2.5.501 17927473

[17] Pitout JD. 2010. Infections with extended-spectrum beta-lactamase-producing enterobacteriaceae: changing epidemiology and drug treatment choices. Drugs 70:313–333. doi:10.2165/11533040-000000000-00000 20166768

[18] Peirano G, Pitout JDD. 2019. Extended-spectrum β-lactamase-producing enterobacteriaceae: update on molecular epidemiology and treatment options. Drugs 79:1529–1541. doi:10.1007/s40265-019-01180-3 31407238

[19] Pitout JDD, Chen L. 2023. The significance of epidemic plasmids in the success of multidrug-resistant drug pandemic extraintestinal pathogenic *Escherichia coli*. Infect Dis Ther 12:1029–1041. doi:10.1007/s40121-023-00791-4 PMC10147871 36947392

[20] Peirano G, Pitout JDD. 2025. Rapidly spreading enterobacterales with OXA-48-like carbapenemases. J Clin Microbiol 63:e0151524. doi:10.1128/jcm.01515-24 PMC11837536 39760498

[21] Pitout JDD, Peirano G, Kock MM, Strydom KA, Matsumura Y. 2019. The global ascendency of OXA-48-type carbapenemases. Clin Microbiol Rev 33:e00102-19. doi:10.1128/CMR.00102-19 PMC6860007 31722889

[22] Mathers AJ, Peirano G, Pitout JDD. 2015. The role of epidemic resistance plasmids and international high-risk clones in the spread of multidrug-resistant enterobacteriaceae. Clin Microbiol Rev 28:565–591. doi:10.1128/CMR.00116-14 PMC4405625 25926236

[23] Pitout JD, Peirano G, DeVinney R. 2023. The contributions of multidrug resistant clones to the success of pandemic extra-intestinal pathogenic *Escherichia coli.* Expert Rev Anti Infect Ther 21:343–353. doi:10.1080/14787210.2023.2184348 36822840

[24] Pitout JDD, Peirano G, Chen L, DeVinney R, Matsumura Y. 2022. *Escherichia coli* ST1193: following in the footsteps of *E. coli* ST131. Antimicrob Agents Chemother 66:e0051122. doi:10.1128/aac.00511-22 PMC9295538 35658504

[25] Scheffers DJ, Pinho MG. 2005. Bacterial cell wall synthesis: new insights from localization studies. Microbiol Mol Biol Rev 69:585–607. doi:10.1128/MMBR.69.4.585-607.2005 PMC1306805 16339737

[26] Sauvage E, Kerff F, Terrak M, Ayala JA, Charlier P. 2008. The penicillin-binding proteins: structure and role in peptidoglycan biosynthesis. FEMS Microbiol Rev 32:234–258. doi:10.1111/j.1574-6976.2008.00105.x 18266856

[27] Zapun A, Contreras-Martel C, Vernet T. 2008. Penicillin-binding proteins and beta-lactam resistance. FEMS Microbiol Rev 32:361–385. doi:10.1111/j.1574-6976.2007.00095.x 18248419

[28] Spratt BG, Cromie KD. 1988. Penicillin-binding proteins of gram-negative bacteria. Rev Infect Dis 10:699–711. doi:10.1093/clinids/10.4.699 3055170

[29] Sethuvel DPM, Bakthavatchalam YD, Karthik M, Irulappan M, Shrivastava R, Periasamy H, Veeraraghavan B. 2023. β-lactam resistance in ESKAPE pathogens mediated through modifications in penicillin-binding proteins: an overview. Infect Dis Ther 12:829–841. doi:10.1007/s40121-023-00771-8 PMC10017896 36877435

[30] Kocaoglu O, Carlson EE. 2015. Profiling of β-lactam selectivity for penicillin-binding proteins in *Escherichia coli* strain DC2. Antimicrob Agents Chemother 59:2785–2790. doi:10.1128/AAC.04552-14 PMC4394777 25733506

[31] Alm RA, Johnstone MR, Lahiri SD. 2015. Characterization of *Escherichia coli* NDM isolates with decreased susceptibility to aztreonam/avibactam: role of a novel insertion in PBP3. J Antimicrob Chemother 70:1420–1428. doi:10.1093/jac/dku568 25634992

[32] Sato T, Yamawaki K. 2019. Cefiderocol: discovery, chemistry, and *in vivo* profiles of a novel siderophore cephalosporin. Clinical Infectious Diseases 69:S538–S543. doi:10.1093/cid/ciz826 PMC6853759 31724047

[33] Chen L, Peirano G, Kreiswirth BN, Devinney R, Pitout JDD. 2022. Acquisition of genomic elements were pivotal for the success of *Escherichia coli* ST410. J Antimicrob Chemother 77:3399–3407. doi:10.1093/jac/dkac329 PMC10205468 36204996

[34] Zhang Y, Kashikar A, Brown CA, Denys G, Bush K. 2017. Unusual *Escherichia coli* PBP 3 insertion sequence identified from a collection of carbapenem-resistant enterobacteriaceae tested *In vitro* with a combination of ceftazidime-, ceftaroline-, or aztreonam-avibactam. Antimicrob Agents Chemother 61:e00389-17. doi:10.1128/AAC.00389-17 PMC5527577 28559260

[35] Ma K, Feng Y, McNally A, Zong Z. 2020. Struggle to survive: the choir of target alteration, hydrolyzing enzyme, and plasmid expression as a novel aztreonam-avibactam resistance mechanism. mSystems 5:e00821-20. doi:10.1128/mSystems.00821-20 PMC7646527 33144312

[36] Freire S, Findlay J, Gruner E, Bruderer V, Nordmann P, Poirel L. 2024. Modification of the penicillin-binding-protein 3 as a source of resistance to broad-spectrum cephalosporins in *Escherichia coli*. J Antimicrob Chemother 79:930–932. doi:10.1093/jac/dkae020 PMC10984938 38305674

[37] Long H, Zhao F, Feng Y, Zong Z. 2025. Global emergence of *Escherichia coli* with PBP3 insertions. J Antimicrob Chemother 80:178–181. doi:10.1093/jac/dkae393 39478333

[38] Sato T, Ito A, Ishioka Y, Matsumoto S, Rokushima M, Kazmierczak KM, Hackel M, Sahm DF, Yamano Y. 2020. *Escherichia coli* strains possessing a four amino acid YRIN insertion in PBP3 identified as part of the SIDERO-WT-2014 surveillance study. JAC Antimicrob Resist 2:dlaa081. doi:10.1093/jacamr/dlaa081 PMC8210206 34223033

[39] Mendes RE, Doyle TB, Streit JM, Arhin FF, Sader HS, Castanheira M. 2021. Investigation of mechanisms responsible for decreased susceptibility of aztreonam/avibactam activity in clinical isolates of enterobacterales collected in Europe, Asia and Latin America in 2019. J Antimicrob Chemother 76:2833–2838. doi:10.1093/jac/dkab279 PMC8561256 34436603

[40] Martin MJ, Luo TL, Kovalchuk V, Kondratiuk V, Dao HD, Kovalenko I, Plaza BJ, Kettlewell JM, Anderson CP. 2024. Detection of cefiderocol and aztreonam/avibactam resistance in epidemic *Escherichia coli* ST-361 carrying bla(NDM-5) and bla(KPC-3) from foreign fighters evacuated from Ukraine. Antimicrob Agents Chemother 68:e0109024. doi:10.1128/aac.01090-24 PMC11539215 39302119

[41] Eklund E, Maruca T, Kruczynski K, Critchfield H, Murphy R, Keller E, Lee B, Patel A, Klein L, Torpey DA, Myers RA, Campodónico VL. 2026. High *in vitro* activity of aztreonam/avibactam against NDM-producing enterobacterales with emerging resistance in *Escherichia coli* in the United States Mid-Atlantic region. Microbiol Spectr 14:e0397125. doi:10.1128/spectrum.03971-25 PMC12955408 41665356

[42] Peirano G, Chen L, Nobrega D, Finn TJ, Kreiswirth BN, DeVinney R, Pitout JDD. 2022. Genomic epidemiology of global carbapenemase-producing *Escherichia coli*, 2015-2017. Emerg Infect Dis 28:924–931. doi:10.3201/eid2805.212535 PMC9045447 35451367

[43] Simner PJ, Bergman Y, Conzemius R, Jacobs E, Tekle T, Beisken S, Tamma PD. 2023. An NDM-producing *Escherichia coli* clinical isolate exhibiting resistance to cefiderocol and the combination of ceftazidime-avibactam and aztreonam: another step toward pan-β-lactam resistance. Open Forum Infect Dis 10:ofad276. doi:10.1093/ofid/ofad276 PMC10319620 37416757

[44] Ma K, Zong Z. 2022. Resistance to aztreonam-avibactam due to CTX-M-15 in the presence of penicillin-binding protein 3 with extra amino acids in *Escherichia coli*. Front Microbiol 13:1047109. doi:10.3389/fmicb.2022.1047109 PMC9674307 36406430

[45] Livermore DM, Mushtaq S, Vickers A, Woodford N. 2023. Activity of aztreonam/avibactam against metallo-β-lactamase-producing enterobacterales from the UK: Impact of penicillin-binding protein-3 inserts and CMY-42 β-lactamase in *Escherichia coli*. Int J Antimicrob Agents 61:106776. doi:10.1016/j.ijantimicag.2023.106776 36893810

[46] Pitout JDD, Peirano G, Matsumura Y, DeVinney R, Chen L. 2024. *Escherichia coli* sequence type 410 with carbapenemases: a paradigm shift within *E. coli* toward multidrug resistance. Antimicrob Agents Chemother 68:e0133923. doi:10.1128/aac.01339-23 PMC10869336 38193668

[47] Bakthavatchalam YD, Abdullah F, Srinivasan D, Nithiyanandam S, Neeravi A, Shah P, Subburaju N, Jaganathan SV, Devi R, Nataraj G, Yesudason BL, Walia K, Veeraraghavan B. 2024. Development and validation of a pentaplex PCR assay for rapid detection of blaCTX-M, blaOXA–1, blaCMY, blaNDM and the PBP3 insert in enterobacterales. Indian J Med Microbiol 52:100710. doi:10.1016/j.ijmmb.2024.100710 39181333

[48] Peirano G, Walker SD, Duong MS, Frank K, Delantar JM, Lisboa L, Chan WW, Pitout JDD. 2026. Aztreonam plus ceftazidime/avibactam broth disk elution test: failure to detect *Escherichia coli* with PBP-3 insertions. Eur J Clin Microbiol Infect Dis 45:1285–1295. doi:10.1007/s10096-026-05406-7 41577890

[49] Papp-Wallace KM, Bonomo RA. 2016. New β-lactamase inhibitors in the clinic. Infect Dis Clin North Am 30:441–464. doi:10.1016/j.idc.2016.02.007 PMC4980828 27208767

[50] Mojica MF, Rossi MA, Vila AJ, Bonomo RA. 2022. The urgent need for metallo-β-lactamase inhibitors: an unattended global threat. Lancet Infect Dis 22:e28–e34. doi:10.1016/S1473-3099(20)30868-9 PMC8266270 34246322

[51] Cercenado E, Candel FJ. 2025. Classification and applicability of new beta-lactamase inhibitors. Rev Esp Quimioter 38 Suppl 1:21–26. doi:10.37201/req/s01.03.2025 PMC12584905 41159226

[52] Katsarou A, Stathopoulos P, Tzvetanova ID, Asimotou CM, Falagas ME. 2025. β-Lactam/β-lactamase inhibitor combination antibiotics under development. Pathogens 14:168. doi:10.3390/pathogens14020168 PMC11858600 40005543

[53] Zhanel GG, Lawson CD, Adam H, Schweizer F, Zelenitsky S, Lagacé-Wiens PRS, Denisuik A, Rubinstein E, Gin AS, Hoban DJ, Lynch JP 3rd, Karlowsky JA. 2013. Ceftazidime-avibactam: a novel cephalosporin/β-lactamase inhibitor combination. Drugs 73:159–177. doi:10.1007/s40265-013-0013-7 23371303

[54] van Duin D, Bonomo RA. 2016. Ceftazidime/avibactam and ceftolozane/tazobactam: second-generation β-lactam/β-lactamase inhibitor combinations. Clin Infect Dis 63:234–241. doi:10.1093/cid/ciw243 PMC4928383 27098166

[55] Bush K. 2025. Past, present, and future perspectives on aztreonam and avibactam. Expert Rev Anti Infect Ther 23:277–290. doi:10.1080/14787210.2025.2473047 40011051

[56] Sargianou M, Stathopoulos P, Vrysis C, Tzvetanova ID, Falagas ME. 2025. New β-lactam/β-lactamase inhibitor combination antibiotics. Pathogens 14:307. doi:10.3390/pathogens14040307 PMC12029989 40333039

[57] Zhanel GG, Lawrence CK, Adam H, Schweizer F, Zelenitsky S, Zhanel M, Lagacé-Wiens PRS, Walkty A, Denisuik A, Golden A, Gin AS, Hoban DJ, Lynch JP 3rd, Karlowsky JA. 2018. Imipenem-relebactam and meropenem-vaborbactam: two novel carbapenem-β-lactamase inhibitor combinations. Drugs 78:65–98. doi:10.1007/s40265-017-0851-9 29230684

[58] Yahav D, Giske CG, Grāmatniece A, Abodakpi H, Tam VH, Leibovici L. 2020. New β-lactam-β-lactamase inhibitor combinations. Clin Microbiol Rev 34:e00115-20. doi:10.1128/CMR.00115-20 PMC7667665 33177185

[59] Lepak AJ, Zhao M, Andes DR. 2019. WCK 5222 (Cefepime/Zidebactam) pharmacodynamic target analysis against metallo-β-lactamase producing enterobacteriaceae in the neutropenic mouse pneumonia model. Antimicrob Agents Chemother 63:e01648-19. doi:10.1128/AAC.01648-19 PMC6879221 31591114

[60] Keam SJ. 2023. Sulbactam/durlobactam: first approval. Drugs 83:1245–1252. doi:10.1007/s40265-023-01920-6 37523122

[61] Andrei S, Valeanu L, Chirvasuta R, Stefan MG. 2018. New FDA approved antibacterial drugs: 2015-2017. Discoveries (Craiova) 6:e81. doi:10.15190/d.2018.1 PMC7086068 32309599

[62] Lee YR, Baker NT. 2018. Meropenem-vaborbactam: a carbapenem and beta-lactamase inhibitor with activity against carbapenem-resistant enterobacteriaceae. Eur J Clin Microbiol Infect Dis 37:1411–1419. doi:10.1007/s10096-018-3260-4 29675787

[63] Zhanel GG, Mansour C, Mikolayanko S, Lawrence CK, Zelenitsky S, Ramirez D, Schweizer F, Bay D, Adam H, Lagacé-Wiens P, Walkty A, Irfan N, Clark N, Nicolau D, Tascini C, Karlowsky JA. 2024. Cefepime-taniborbactam: a novel cephalosporin/β-lactamase inhibitor combination. Drugs 84:1219–1250. doi:10.1007/s40265-024-02082-9 39214942

[64] Wagenlehner FM, Gasink LB, McGovern PC, Moeck G, McLeroth P, Dorr M, Dane A, Henkel T, Team C-S. 2024. Cefepime-taniborbactam in complicated urinary tract infection. N Engl J Med 390:611–622. doi:10.1056/NEJMoa2304748 38354140

[65] Tamma PD, Munita JM. 2024. The metallo-β-lactamases strike back: emergence of taniborbactam escape variants. Antimicrob Agents Chemother 68:e0151023. doi:10.1128/aac.01510-23 PMC10848767 38174925

[66] Le Terrier C, Drusin SI, Nordmann P, Pitout J, Peirano G, Vila AJ, Moreno DM, Poirel L. 2025. The emerging concern of IMP variants being resistant to the only IMP-type metallo-β-lactamase inhibitor, xeruborbactam. Antimicrob Agents Chemother 69:e0029725. doi:10.1128/aac.00297-25 PMC12217461 40488615

[67] Mataseje LF, Chen L, Peirano G, Fakharuddin K, Kreiswith B, Mulvey M, Pitout JDD. 2022. Klebsiella pneumoniae ST147: and then there were three carbapenemases. Eur J Clin Microbiol Infect Dis 41:1467–1472. doi:10.1007/s10096-022-04514-4 36271303

[68] Comini S, Boattini M, Gaibani P, Bianco G. 2026. Cefepime combined with late-generation β-lactamase inhibitors: mechanisms of action, *in vitro* activity, pk/pd characteristics, clinical evidence and resistance mechanisms. Antibiotics (Basel) 15:263. doi:10.3390/antibiotics15030263 PMC13024656 41892425

[69] Le Terrier C, Nordmann P, Sadek M, Poirel L. 2023. *In vitro* activity of cefepime/zidebactam and cefepime/taniborbactam against aztreonam/avibactam-resistant NDM-like-producing *Escherichia coli* clinical isolates. J Antimicrob Chemother 78:1191–1194. doi:10.1093/jac/dkad061 PMC10154122 36921067

[70] Sánchez-Peña L, Rodríguez-Pallares S, Aja-Macaya P, Blanco-Martín T, González-Pinto L, Pérez-Rodríguez G, Le Terrier C, Portillo-Calderón I, Recacha E, Riazzo C, Vázquez-Ucha JC, Beceiro A, Aracil B, Oteo-Iglesias J, Martínez-Martínez L, Poirel L, Bou G, Arca-Suárez J. 2025. Broad spectrum of β-lactamase coverage and potent antimicrobial activity of xeruborbactam in combination with meropenem against carbapenemase-producing enterobacterales, including strains resistant to new β-lactam/β-lactamase inhibitor combinations. Antimicrob Agents Chemother 69:e0053325. doi:10.1128/aac.00533-25 PMC12406686 40711423

[71] Mushtaq S, Vickers A, Woodford N, Livermore DM. 2024. Activity of aztreonam/avibactam and ceftazidime/avibactam against enterobacterales with carbapenemase-independent carbapenem resistance. Int J Antimicrob Agents 63:107081. doi:10.1016/j.ijantimicag.2023.107081 38176458

